# Roles of Melatonin in Fetal Programming in Compromised Pregnancies

**DOI:** 10.3390/ijms14035380

**Published:** 2013-03-06

**Authors:** Yu-Chieh Chen, Jiunn-Ming Sheen, Miao-Meng Tiao, You-Lin Tain, Li-Tung Huang

**Affiliations:** 1 Department of Pediatrics, Kaohsiung Chang Gung Memorial Hospital, Chang Gung University College of Medicine, Kaohsiung 833, Taiwan; E-Mails: gesicht27@gmail.com (Y.-C.C.); ray.sheen@gmail.com (J.-M.S.); pc006581@yahoo.com.tw (M.-M.T.); tainyl@hotmail.com (Y.-L.T.); 2 Center for Translational Research in Biomedical Sciences, Kaohsiung Chang Gung Memorial Hospital, Chang Gung University College of Medicine, Kaohsiung 833, Taiwan; 3 Department of Traditional Chinese Medicine, Chang Gung University, Linkow 333, Taiwan

**Keywords:** melatonin, epigenetic, fetal programming, redox, pregnancy

## Abstract

Compromised pregnancies such as those associated with gestational diabetes mellitus, intrauterine growth retardation, preeclampsia, maternal undernutrition, and maternal stress may negatively affect fetal development. Such pregnancies may induce oxidative stress to the fetus and alter fetal development through the epigenetic process that may affect development at a later stage. Melatonin is an oxidant scavenger that reverses oxidative stress during the prenatal period. Moreover, the role of melatonin in epigenetic modifications in the field of developmental programming has been studied extensively. Here, we describe the physiological function of melatonin in pregnancy and discuss the roles of melatonin in fetal programming in compromised pregnancies, focusing on its involvement in redox and epigenetic mechanisms.

Abbreviations

AGE: Advanced glycation end product
CREB: cAMP response element-binding protein
DNMT1: DNA (cytosine-5)-methyltransferase 1
GDM: gestational diabetis mellitus
GR: glucocorticoid receptor
IUGR: intrauterine growth retardation
MeCP2: methyl CpG binding protein 2
mGlu: metabotropic glutamate receptor
MT: melatonin receptor
NF-E2-related factor: nuclear factor erythroid 2-related factor 2
NF-κB: nuclear factor kappa-light-chain-enhancer of activated B cells
Pdx-1: pancreatic and duodenal homeobox 1
Per1: period circadian protein homolog 1
PPAR: peroxisome proliferator-activated receptor
ROS: reactive oxygen species
REM: rapid eye movement
SCN: suprachiasmatic nucleus

## 1. Introduction

Melatonin (*N*-acetyl-5-methoxytryptamine), an endogenously produced indoleamine of the pineal gland, is an antioxidant, free radical scavenger, anti-inflammatory molecule, and is produced and secreted in a circadian fashion [1–4]. Melatonin was first identified during the late 1950s by Lerner *et al.* in the pineal gland [5]. It was first identified and named for its skin-lightening properties observed in fish and frog melanocytes. Melatonin is a small lipophilic indoleamine that crosses the placenta and blood-brain barrier and enters cells rapidly. Melatonin acts as a free radical scavenger and protects nuclear and mitochondrial DNA from the damage induced by free radicals [6]. In addition, melatonin stimulates the expression of antioxidant enzyme [7,8] and anti-inflammatory genes [4]. Recent studies involving the role of melatonin in the epigenetic modifications associated with developmental programming are emerging rapidly [8,9]. Moreover, melatonin can stimulate the immune system, protecting organisms against bacterial and viral infections [1,2].

Melatonin has been used clinically in cancer [10], neurodegenerative diseases [11,12], sleep disorders [13,14], aging [11,15], and in neonatal and pediatric diseases of neonates and children [16,17]; however, its utility in pregnancy has rarely been discussed [17–21].

## 2. Melatonin Synthesis and Its Receptors

In mammals, the melatonin rhythm is generated by an endogenous circadian clock in the suprachiasmatic nucleus (SCN) of the hypothalamus [22]. An individual must predict the upcoming seasonal changes to adapt both physiological and behavioral functions. The biosynthesis and metabolism of melatonin are regulated by the light/dark cycle. Once synthesized, melatonin is not stored in pineal cells but is rapidly released into the bloodstream. Circulating melatonin provides circadian and seasonal timing cues. Melatonin is locally found in various cells, tissues, and organs, including lymphocytes, bone marrow, thymus, gastrointestinal tract, skin, the Harderian gland, and the retina [1–3]. Melatonin production has also been detected in invertebrates, bacteria, and plants [22].

Melatonin is synthesized primarily in the pineal gland from the amino acid precursor tryptophan. At least four enzymes are involved in melatonin biosynthesis, among which serotonin *N*-acetyltransferase is considered the rate-limiting enzyme. The half-life of melatonin in serum varies between less than 30 min and 60 min. Seventy percent of serum melatonin is bound to albumin, and the remaining 30% diffuses in the surrounding tissues [23]. It is metabolized primarily in the liver and secondarily in the kidney [22]. Melatonin is catabolized to 6-sulfoxy-melatonin exclusively by hepatic P450 monooxygenase; after conjugation, 6-sulfoxy melatonin then forms the urinary 6-sulfoxy-melatonin [23,24].

Two types of membrane-associated melatonin receptors have been cloned in mammals [25]; these receptors, MT1 and MT2, which belong to the G-protein-coupled receptor superfamily, have seven transmembrane domains. The MT1 and MT2 melatonin receptors show 60% homology at the amino acid level. A third membrane-associated receptor, MT3, has been identified as the quinone reductase 2. MT3 belongs to a group of reductases that are involved in the protection against oxidative stress by preventing the electron transfer reactions of quinones.

## 3. Melatonin Possesses Both Antioxidant and Epigenetic Modifications Abilities

Melatonin has pleiotropic bioactivities and is involved in the regulation of the circadian rhythm, reproductive physiology, anti-inflammation, sleep promotion, and body temperature regulation [2,3]. In addition, melatonin and its metabolites have been reported to have important antioxidant properties owing to their direct and indirect antioxidant activities [2,3].

Oxidative stress is defined as an imbalance between prooxidant and antioxidant forces leading to an overall prooxidant insult. Melatonin and its byproducts are capable of scavenging both ROS and reactive nitrogen species (RNS), including the hydroxyl radical (•OH), hydrogen peroxide (H_2_O_2_), singlet oxygen (^1^O_2_), hypochlorous acid (HClO), peroxynitrite anion (ONOO^−^), and/or peroxynitrous acid (ONOOH) [11]. Melatonin can protect both humans and animals from oxidative stress [26,27].

Melatonin also acts as a potent endogenous antioxidant by scavenging free radicals and upregulating antioxidant pathways. The activity and expression of antioxidant enzymes such as superoxide dismutase, glutathione, catalase, glutathione peroxidase, and glutathione reductase are increased by melatonin, which supports data concerning its indirect antioxidant action [11]. Notably, melatonin upregulates superoxide dismutase 2 mRNA expression via an epigenetic mechanism [9]. Further evidence of the antioxidant effect of melatonin is provided by its ability to reduce lipid peroxidation, a degradative phenomenon involved in the pathogenesis of many diseases [11].

Melatonin is a much more potent antioxidant than many traditionally used antioxidants and has two unique features. First, melatonin does not undergo redox cycling and once oxidized, cannot be regenerated to its reduced form [28]. Second, the antioxidant action of melatonin involves the donation of two electrons instead of one; therefore, melatonin does not become a free radical in the antioxidant process [28]. Furthermore, melatonin can be metabolized to both kynurenic acid and to *N*^1^-acetyl-*N*^2^-formyl-5-methoxykynuramine and *N*^1^-acetyl-5-methoxykynuramine [26]. These metabolites are very powerful antioxidants and cyclooxygenase-2 inhibitors. Therefore, they are considered as potentially selective anti-inflammatory agents [1–3].

Mitochondria produce high amounts of ROS during their biogenesis. Mitochondria, which are characterized by a double membrane, contain several hundreds of proteins and 2–10 copies of mitochondrial DNA in the matrix enclosed by the mitochondrial inner membrane. Mitochondria play a critical role in generating energy in most eukaryotic cells. In this context, melatonin can stimulate mitochondrial biogenesis and increase the efficiency of the electron transport chain, thereby limiting electron leakage and free radical generation. In addition, the antioxidant effect of melatonin and its ability to increase mitochondrial glutathione levels is of great importance for mitochondrial physiology [29].

Epigenetic modifications refer to stable and heritable gene expression changes that are not mediated by DNA sequence alterations. Unlike genetic information, which is stable, epigenetic modifications occur in response to early environmental signals and instantly respond to transient stimuli, resulting in modified gene expression patterns and phenotypes later in life. Melatonin may regulate antioxidant gene and enzyme expression through epigenetic mechanisms [9]. Melatonin induces the expression of the *Nrf2* gene and suppresses that of NF-κB through epigenetic processes [9]. In addition, certain anti-cancer effects of melatonin are the result of its effect on epigenetic changes [30].

## 4. Physiological Functions of Melatonin during Pregnancy

Melatonin plays an important role in pregnancy and parturition [18,19]. Maternal plasma melatonin levels are elevated during pregnancy, reaching a maximum at term, and returning to basal levels immediately after delivery [18]. The placenta expresses melatonin receptors and melatonin easily crosses the placenta without being altered [18]. During normal pregnancy, melatonin acts as an antioxidant and appears to be essential for a successful pregnancy. Sandyk *et al.* had found the association of melatonin and spontaneous abortion which was presumed to be due to melatonin’s role in diminishing uterine contractions by decreasing the production of prostaglandins and in prevention of the immunologic rejection of trophoblast by stimulating the progesterone production [31]. Further, Matsuzuka *et al.* showed that alleviation of the embryo death might be achieved by administration of melatonin in mice [32]. Moreover, maternal melatonin also plays a key role in the regulation of development of fetal organs that are critical for the successful adaptation of the neonate to extrauterine life [33].

In rodents, melatonin-binding sites are observed in the fetal pituitary gland by gestational day 15 and in the SCN by gestational day 18 [34,35]. Melatonin receptors are present in the human fetal SCN [36] and in many areas of the fetal human brain [37]. Therefore, maternal melatonin may be involved in various fetal functions.

Predicting the upcoming seasons to adapt physiological and behavioral functions is important for the survival of individuals and the perpetuity of species. Information about day length and circadian phase is transferred to the fetus prenatally [38]. Maternal melatonin crosses the placenta freely and enters the fetal circulation easily, playing a critical role in providing photoperiodic information to the fetus. In sheep and ewes, reproductive activity is initiated during the fall and inhibited during summer; in such organisms, melatonin has a stimulatory effect on the reproductive axis and influences the photoperiod through pulsatile secretion of luteinizing hormone [39]. This has been observed after the removal of the pineal gland, which disrupts the photoperiod-induced reproductive responses to seasonal changes according to the duration of day and night [40].

During pregnancy, circadian variations in melatonin levels in the maternal circulation have been reported in sheep [41,42] and rats [43]. The passage of maternal melatonin through the placenta exposes the fetus to a daily melatonin rhythm of low concentrations during the day and high concentrations at night [44,45]. The maternal melatonin circadian rhythm is linked to the generation of the circadian rhythms in the fetal adrenal gland [38].

Torres-Farfan *et al.* reported that maternal melatonin decreased cortisol production in the fetal adrenal gland of the capuchin monkey [46]. In a subsequent study on sheep, they found that melatonin had direct inhibitory effects on noradrenaline-stimulated fetal cerebral artery contraction, the release of glycerol by brown adipose tissue, and on ACTH-induced secretion of cortisol by the fetal adrenal gland [33]. The lack of maternal melatonin during the early stages of gestation was found to disrupt drinking behavior of rat pups, and this effect is reversed by the administration of exogenous melatonin to the mother [47]. In addition, low levels or a lack of a circadian rhythm of fetal corticosterone may result in intrauterine growth retardation [48]. Taken together, these findings indicate that maternal melatonin plays a key role in both the regulation of the development of fetal organs critical for the successful adaptation of the neonate to extrauterine life and prevention of pregnancy loss.

## 5. Safety Profiles and Side Effects of Melatonin in Pregnancy

There is general agreement that melatonin therapy has a remarkably benign safety profile. Melatonin has shown no obvious detrimental effects on mouse and rat embryo development in toxicity tests [49,50]. In pregnant rats, administration of high doses of melatonin (200 mg·kg^−1^·day^−1^) from gestational days 6–19 did not adversely affect the development of rat pups [51]. In a study by Sadowsky *et al.*, high-dose melatonin had no apparent effect on fetal or maternal well-being, and it did not affect myometrial activity during late gestation [52]. However, melatonin has been shown to inhibit the activity of prostaglandin synthases, and prostaglandins have important circulatory and endocrine functions in the fetus [53].

## 6. The Concept of Fetal Programming

The plasticity of the developmental process allows the organism to respond to the surrounding environment. Programming is defined as the induction, silencing, or restriction of the development of somatic structures or a physiological system, which results in long-term effects. Human epidemiological studies have provided convincing support for the concept of developmental programming by showing a strong association between low birth weight and an increased risk of adverse outcomes in adulthood, such as coronary heart disease, stroke, high blood pressure, and type 2 diabetes [54].

Perturbations of the developmental adaptation process can also have adverse consequences on organ function and disease risk later in life. The placenta plays a critical role in fetal programming. Maternal complications of pregnancy such as gestational diabetes mellitus (GDM), intrauterine growth restriction (IUGR), preeclampsia, maternal undernutrition, and maternal stress are associated with an increased risk of brain dysfunction, cardiovascular disease, and metabolic syndrome in the offspring through fetal programming mechanisms [55–60]. It is now evident that epigenetic regulation plays a critical role in developmental programming.

## 7. Redox and Epigenetic Mechanisms in Fetal Programming

Several mechanisms contribute to fetal programming. In this review, we discuss two important mechanisms underlying fetal programming, redox and epigenetic alterations [55–60]. Detailed reviews of the mechanisms of fetal programming can be found in the literature [61,62].

### 7.1. The Role of Redox Alterations in Fetal Programming

ROS are produced within the follicle; these play a physiological role in the process of ovulation [63]. Pregnancy is associated with physiologically increased oxidative stress in the mother [19,64]. The placenta is of utmost importance for intrauterine fetal development and growth and is susceptible to oxygen tension changes [65].

Extensive increases in oxidative stress have been observed in association with various conditions, including smoking, diabetes, hypertension, and hypoxia, which are not uncommon in pregnant women. These conditions can cause increased oxidative stress either in the mother or the fetus, which may result in the programming of diseases in the offspring at a later stage [58,66].

### 7.2. The Role of Epigenetic Modifications in Fetal Programming

Epigenetic modifications refer to stable and heritable gene expression changes without alterations of the DNA sequence. The mechanisms of epigenetic change include posttranslational histone modifications and DNA methylation, imprinting, and small-RNA mediated controls. Epigenetic modifications perceive the effect of early environmental signals and play a role in programming.

In the first stages of placental vascular development, endothelial specialization and blood vessel formation are controlled by epigenetic mechanisms [67]. Moreover, the expression of genes implicated in trophoblast invasion, such as maspin, is regulated by histone modifications [68]. Recent studies have shown that placental dysfunction owing to maladaptation to external stressors during pregnancy plays a critical role in fetal programming, as evidenced by changes in the placental size, molecular components, and histopathology, which may result in dismal maternal and fetal outcomes [69]. Deregulation of placentation can lead to adverse outcomes for both the mother and fetus, as evidenced by alterations of the epigenetic profile in cultured human trophoblasts [70]. Taken together, these findings indicate that environmental cues *in utero* might produce long-term consequences through the epigenetic mechanism [71].

### 7.3. Redox Alterations in Utero Play a Role in Epigenetic Modifications

Increased levels of ROS are important in epigenetic modification of DNA or chromatin and influences gene expression and cell differentiation [72,73]. The effect of ROS on epigenetic alterations has been documented in cancer studies [74]. The glucocorticoid receptor (GR) is susceptible to redox influences and epigenetic modifications and is therefore an example of the involvement of oxidative stress in epigenetic modifications and programming [59].

## 8. The Roles of Melatonin in Redox and Epigenetic Alterations in Fetal Programming

### 8.1. Melatonin Has a Role in Redox Modifications in Fetal Programming

Human follicular fluid contains high concentrations of melatonin [75]. Melatonin has a direct role in oocyte maturation and embryo development because it decreases oxidative stress in ovarian follicles and protects oocytes from free radical damage [65]. In addition, melatonin can increase glutathione peroxidase activity in the human chorion [76].

Shift work is common during pregnancy and may disrupt the maternal melatonin rhythm. Epidemiological studies in women show that shift work increases the risk of premature delivery and the incidence of low birth weight [77,78], which are both strongly related to redox-related fetal programming. In animal studies, the suppression of maternal melatonin inhibited fetal adrenal maturation with a subsequent IUGR [48] and glucose intolerance in the offspring [79]. Melatonin thus plays a role in redox regulation and fetal programming.

### 8.2. Melatonin Has a Role in Epigenetic Modifications in Fetal Programming

Exposure of dams to a high-fat diet has been shown to increase fetal liver lipid accumulation and to increase the expression of genes involved in the liver gluconeogenic pathway [80,81]. Alterations in the expression of the clock genes *Per1* and *Npas2* are caused by increased occupancy of H3K14 at the histone acetylase sites in the *Npas2* promoter; these alterations suggested that melatonin plays a role in epigenetic programming [80,81].

Melatonin has been suggested to regulate the expression of antioxidant genes and enzymes through epigenetic mechanisms [9]. Sun *et al.*[82] showed that both its gene expression and Nrf2-dependent antioxidant enzyme expression are dependent on Nrf2 acetylation by CBP/p300. Kawai *et al.*[83] demonstrated that acetylation and deacetylation of Nrf2 regulate its transcriptional activity. Therefore, melatonin has the dual role of regulating *Nrf2* gene induction by acetylation and recruiting the basal transcriptional machinery to the promoter region of Nrf2-related genes. Therefore, melatonin may play a role in epigenetic modifications in fetal programming through the regulation of antioxidant enzymes [9].

## 9. The Roles of Melatonin in Compromised Pregnancies

Pregnancy is a physiological state accompanied by a high metabolic demand and elevated requirements for oxygen and hence prone to oxidative stress-induced organ damage. Furthermore, the placenta is a major source of oxidative stress because it is rich in polyunsaturated fatty acids. As gestation progresses, there is a gradual favoring of antioxidant activity over oxidant activity. In parallel, maternal plasma melatonin levels increase during pregnancy, reaching a maximum at term.

**Figure 1:**
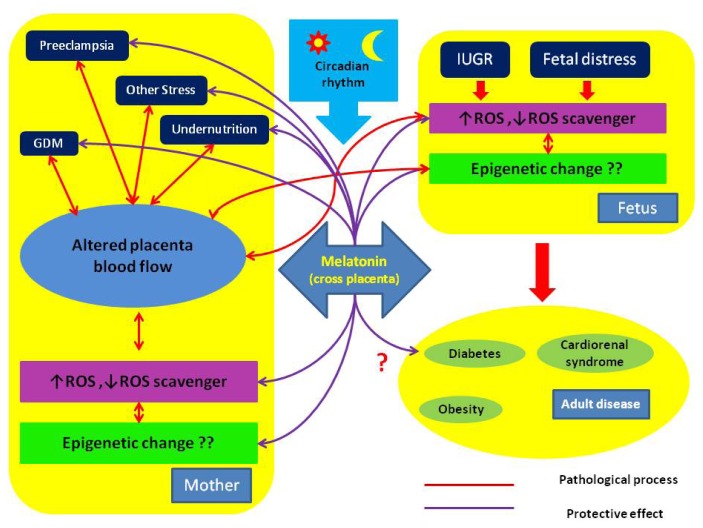
The proposed pathways showing how melatonin can affect normal and compromised pregnancies and result in different adult phenotypes. Melatonin can reduce ROS in both mother and fetus and alters fetal programming in compromised pregnancies through epigenetic changes. GDM (gestational diabetes mellitus); IUGR (intrauterine growth retardation); ROS (reactive oxygen species).

Fetal organs, especially the SCN, are vulnerable to environmental insults via the mother [33,46,84]. In compromised pregnancies, melatonin homeostasis between mother and fetus may be affected [19,33,84]. Furthermore, environmental cues during early development may influence the circadian clock system, which consists of oscillating molecular pacemakers in the hypothalamus, most peripheral tissues, and the hypothalamic-pituitary-adrenal axis and therefore affect the responses to environmental challenges in adult life [85,86]. In addition, premature infants have altered circadian rhythmicity as compared with full-term infants [87,88]. Taken together, these findings indicate that melatonin is involved in fetal programming in compromised pregnancies. Figure 1 depicts the role of melatonin in modulation of pregnancy and fetal programming.

### 9.1. Melatonin and GDM

GDM is a syndrome characterized by glucose intolerance leading to maternal hyperglycemia, endothelial dysfunction, and abnormal regulation of vascular tone [89]. Depending on the diagnostic and screening criteria, the prevalence of gestational diabetes has been reported to range from 1.3% to 19.9% [90]. Placentas from GDM pregnancies are larger than normal [91] and show decreased formation of terminal villi and increased numbers of intermediate villi compared to those from normal pregnancies [92]. These vascular changes are likely to affect placental vascular resistance and vascular volume, leading to metabolic changes in the feto-placental microvascular and macrovascular endothelium [93].

Hyperglycemia increases the activity of the polyol pathway, which decreases antioxidant defenses and increases oxidative stress [94]. Oxidative stress is also increased in the hyperglycemic state by increased glucose auto-oxidation and protein glycation, which upregulate the production of oxidative factors [95]. ROS formation caused by the hyperglycemic state is associated with the progression of vascular complications [96]. ROS can activate numerous pathways that damage cells, and these pathways are often linked to complications that occur in the later stages of diabetes. Brownlee *et al.* reported that hyperglycemia may result in the non-enzymatic glycation of proteins called advanced glycation end products (AGEs), which can interfere with signal transduction and thus change the soluble levels of cytokines, hormones, and free radicals, and these proteins can alter the function of the glycated proteins [97].

In addition to its function as a ROS scavenger involving the amelioration of oxidative damage and the proinflammatory state present in high-risk pregnancies, recent studies have shown that melatonin also plays an important role in the regulation of body weight and adiposity, which could be related to its involvement in the control of the circadian rhythm [98,99]. Moreover, disruption or alterations in endogenous melatonin secretion by the pineal gland have been found to be related to disturbances in glucose and lipid homeostasis [99]. Similarly, genetic variants of the *MTNR1B* gene, a functional melatonin receptor, have been reported to be associated with gestational glucose intolerance in the Chinese population [100]. Therefore, melatonin may function as an antioxidant as well as a metabolic modulator in the context of GDM.

GDM may predispose offspring to many disorders such as obesity, type 2 diabetes mellitus, and cardiorenal metabolic syndrome. The underlying pathophysiology may include epigenetic modifications and alterations in the balance between glucose, insulin, and other regulatory hormones involved in glucose homeostasis during intrauterine and perinatal life [101]. In fetal metabolic programming, an imbalance between leptin and adiponectin leads to obesity, while altered beta-cell proliferation and compensatory islet leads to type 2 diabetes, and beta-cell remodeling and endothelial cell dysfunction leads to cardiorenal syndrome [102]. Pdx-1 is a pancreatic and duodenal homeobox 1 transcription factor that regulates pancreatic development and cell differentiation. There is increased methylation of the CpG island proximal promoter of the *Pdx-1* gene and a subsequent blunting of its transcription, and the development of diabetes during adulthood, explaining an epigenetic mechanism for fetal programming. Recently, Miehle *et al.* conducted a human study to find an association between the percentage of DNA methylation of the leptin gene in the placenta and glycemia using the 2 h post-oral glucose tolerance test at 24–28 weeks of pregnancy [103]. In the same study, DNA methylation in the placenta was inversely correlated with the placental leptin mRNA levels and serum leptin levels in the mother [103]. However, little is known about the role of melatonin in epigenetic modifications in the context of GDM, which warrants further investigation.

### 9.2. Melatonin and Intrauterine Growth Restriction

IUGR is defined as a condition in which the fetus has an estimated body weight and/or length below the 10th percentile for gestational age [104]. IUGR affects most organ systems by interrupting developmental processes and is associated with insulin resistance [105], obesity [106], and cardiovascular disease [107] in adulthood.

Women diagnosed with IUGR show increased values of the indices of oxidative stress in the serum, suggesting the presence of oxidative stress [108,109]. During follow-up, children born with growth retardation show increased levels of lipid peroxidation and have higher blood pressure than age-matched children of normal birth weight [110]. Pregnant rats fed a low-protein diet have offspring with elevated arterial blood pressure and increased vasoconstrictor responsiveness to angiotensin II [111]. Circulating melatonin in pregnant animals was shown to be affected by IUGR [112]. Using a mid- to late-gestation ovine model of IUGR, Lemley *et al.* showed that melatonin might negate the consequences of IUGR in the presence of specific abnormalities in umbilical blood flow as long as sufficient uterine blood perfusion is maintained during pregnancy [113]. Therefore, melatonin may work as an antioxidant in the context of IUGR.

IUGR has been shown to induce epigenetic modification of selected genes in the placenta [114], as well as the liver [115,116], heart [117], pancreas [118], adrenal gland [119], and pulmonary arteries [120]. In rats with IUGR, global decreases in DNA methylation and increases in histone H3 acetylation on lysine 9 (K9) and K14 are observed in the brain at birth [121]. These epigenetic changes are associated with a concomitant decrease in DNMT1, MeCP2, and HDAC1 protein expressions [121]. IUGR in rats induces histone code modifications affecting glut4 expression in skeletal muscle [122], causes increased acetylation of H3K9 and K14 [115], and reduced expression of DNMT1 in the liver [116]. However, little is known about the role of melatonin in epigenetic modifications in the context of IUGR, which warrants further investigation.

### 9.3. Melatonin and Preeclampsia

Preeclampsia is a multisystem disorder that is unique to human pregnancy, occurring in 5%–10% of pregnancies and is a leading cause of maternal and neonatal mortality and morbidity [123]. There is growing evidence that the physiologically immature fetus is highly susceptible to disruptions in placental blood flow, which may predispose an individual to an increased risk of disease beyond the immediate postnatal period. Epidemiological studies show that exposure of infants to preeclampsia during gestation is associated with an increased risk of diabetes and cardiovascular morbidity in adulthood [124].

In women with preeclampsia, lipid peroxide levels in maternal blood and placental tissue are significantly increased. In addition, total antioxidant activities are decreased. Hence, preeclampsia might be considered as an oxidative stress disorder during pregnancy [125]. Endogenous melatonin level is significantly decreased in women with severe preeclampsia [126], who also show alterations in placental melatonin production and melatonin receptor expression [127]. During normal pregnancy, melatonin directly functions as a free radical scavenger and indirectly functions as an antioxidant, and it appears to be essential for successful pregnancy. Besides, melatonin can be a desirable component of the antioxidant system in the human placenta because it significantly improves mitochondrial efficiency [128]. Therefore, melatonin may function as an antioxidant in the context of preeclampsia.

Maternal adversities leading to dysregulation of placental development originated from preimplantation affecting the course of pregnancy were shown in both animal and human studies to alter the epigenetic process (e.g., DNA methylation, histone modifications, and genome imprinting) and result in preeclampsia and dismal fetal outcomes [66,69]. A recent study showed altered global DNA methylation patterns in preeclampsia placentas and its association with blood pressure [129]. Chim *et al.* reported increased concentrations of unmethylated maspin concentrations in the plasma of women with preeclampsia compared with that of healthy pregnant controls [130]. However, little is known about the role of melatonin in epigenetic modifications in the context of preeclampsia, which should be studied further.

### 9.4. Melatonin and Maternal Undernutrition

Adequate nutrition during gestation is essential for fetal growth and development. Maternal undernutrition can significantly affect fetal growth and intrauterine programming. The placenta may act as a nutrient sensor, modifying nutrient and hormone availability to feto-placental tissues in relation to environmental challenges. There is growing evidence linking slow fetal growth with the developmental programming of cardiovascular and metabolic diseases and neuropsychiatric disorders [131].

Maternal undernutrition during pregnancy can result in asymmetric growth retardation and is associated with increased oxidative stress. In humans, fetal undernutrition is associated with significant oxidative stress in small-for-gestational-age neonates born at term to malnourished mothers [132]. Melatonin treatment in undernourished mothers during pregnancy has been shown to improve birth weight and protect the placenta from ischemia/reperfusion-induced oxidative stress [133,134]. Maternal dietary protein restriction during pregnancy was found to have adverse effects on the quality of the sleep-wake cycle in the adult rat offspring [135]. In rat offspring of mothers exposed to a low-protein diet, antenatal administration of antioxidants to the mother prevents the development of hypertension and vascular dysfunction during adulthood [111]. Therefore, melatonin may function as an antioxidant in the context of maternal undernutrition.

Maternal undernutrition can have long-lasting effects on gene expression in the fetus and therefore can extensively affect the phenotypic outcome of the progeny. Maternal undernutrition can result in marked epigenetic changes affecting the GR and proopiomelanocortin (*POMC*) gene expression in the fetal hypothalamus and contributes to fetal programming. The consequences of maternal undernutrition include altered regulation of food intake, energy expenditure, and glucose homeostasis later in life [136]. Studies have shown that modest dietary protein restriction during pregnancy induces an altered phenotype through epigenetic changes in specific genes. Decreased methylation of the GR and PPARα promoters was detected in the heart of the offspring [137] and the PPARα promoter was hypomethylated in the umbilical cord [138]. However, there are a few reports on the role of melatonin in epigenetic modifications in the context of maternal undernutrition and this requires further investigation.

### 9.5. Melatonin and Maternal Stress

The rat model of maternal stress is used to replicate putative factors implicated in the etiology of major depression [139]. It is well known that prenatal exposure to glucocorticoids and stress leads to programming of the hypothalamic-pituitary-adrenal function and behavior and has long-term effects on the offspring [59,139,140]. The effects of prenatal stress on fetal outcome are mediated in part by elevated fetal glucocorticoid exposure.

The maternal milieu may perturb the development of the fetal circadian clock through its effect on glucocorticoid receptors, which are already present in the SCN during early development [141]. Dugovic *et al.* reported that the offspring of stressed mothers showed increased rapid eye movement (REM), total sleep, and an increase in slow-wave sleep during the dark phase [142]. Agomelatine is a mixed MT1/MT2 melatonin receptor agonist and 5HT2C serotonin receptor antagonist. Mariesse *et al.* showed reduced duration of slow-wave sleep, increased duration of REM sleep, increased number of REM sleep events, and increased motor activity before the beginning of the dark phase of the light/dark cycle in adult offspring of mothers exposed to stress [143]. This report provided evidence that agomelatine corrects sleep architecture and restores circadian homeostasis in a rat model of maternal stress [143]. Stress can affect sleep behaviors through its effect on inflammatory cytokines [144], which are susceptible to redox alteration [145,146]. In addition, the anti-inflammatory properties of melatonin arise from the fact that it prevents the translocation of NF-κB to the nucleus, thus reducing the upregulation of proinflammatory cytokines [6]. Taken together, these findings indicate that sleep behavior is affected in the offspring of mothers exposed to stress. Melatonin plays a role in reverting sleep disorders in maternal stress offspring, possibly through the modulation of proinflammatory cytokines via a redox mechanism. The possible role of melatonin in other long-term sequelae in maternal stress offspring warrants further studies.

The effect of maternal stress on fetal programming may be mediated by epigenetic mechanisms, with resulting behavioral modifications and altered biological rhythms in adult offspring [147]. Morley-Fletcher *et al.* demonstrated that agomelatine could correct all biochemical, cellular, and behavioral abnormalities displayed by maternal stress rats in adult life [148]. These authors showed that agomelatine reversed the reduction in the levels of p-CREB, mGlu2/3 receptors, and mGlu5 receptors in the hippocampus of maternal stress rats [148]. Interestingly, the mGlu2/3 receptor was shown to be altered via an epigenetic mechanism in maternal stress adult offspring [149], suggesting that melatonin has a potential role in epigenetic modifications in the context of maternal stress.

## 10. Conclusions

Melatonin is a potent free radical scavenger, a broad-spectrum antioxidant, and an epigenetic modification agent. The role of melatonin in pregnancy and parturition is well established. Melatonin readily crosses the placenta and the fetal blood-brain barrier and plays a key role in the regulation of development of fetal organs to extrauterine life. Compromised pregnancies result in oxidative stress to the fetus and alter fetal development through the epigenetic process. In this regard, melatonin is beneficial for reversing the adverse programming effects associated with compromised pregnancies via a redox mechanism; however, the potential role of melatonin in epigenetic modifications requires further study. Additional studies exploring the role of melatonin as a target for other pregnancy-related diseases are warranted.
